# Targeting the innate immune receptor TLR8 using small-molecule agents

**DOI:** 10.1107/S2059798320006518

**Published:** 2020-06-17

**Authors:** Kentaro Sakaniwa, Toshiyuki Shimizu

**Affiliations:** a Graduate School of Pharmaceutical Sciences, The University of Tokyo, Hongo, Bunkyo-ku, Tokyo 113-0033, Japan

**Keywords:** Toll-like receptors, innate immunity, structural biology, agonist, antagonist

## Abstract

Toll-like receptors (TLRs) are pattern-recognition receptors that initiate innate immune responses. Among the TLRs, TLR8 (and TLR7) recognizes single-stranded RNA to mediate downstream signals. In recent years, intensive X-ray crystal structural analyses have provided atomic insights into structures of TLR8 complexed with various agonists or antagonists. Here, structural knowledge of the activation and inactivation mechanisms of the ligands is reviewed. In addition, the potential clinical applications of TLR ligands are examined.

## Abstract 2

The innate immune receptor TLR8 can be positively or negatively regulated by small chemical ligands. Structural views of agonist-bound and antagonist-bound forms have revealed the mechanisms underlying agonism and antagonism.

## 1. Introduction   

**Figure 1:**
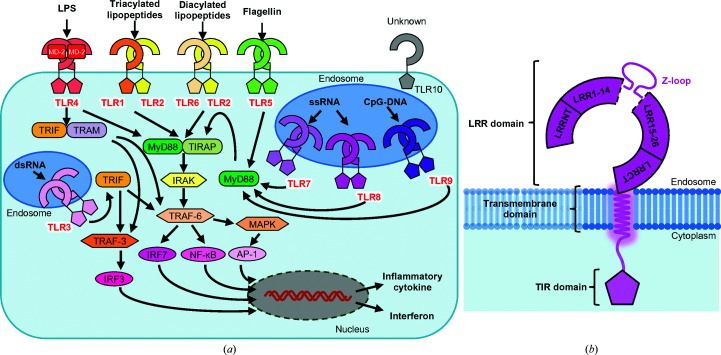
TLR signaling and domain organization of TLR8. (*a*) Overview of TLR ligand and signaling pathways. All TLRs except for TLR3 transduce signals through the MyD88-dependent pathway, and TLR3 and TLR4 transduce signals through a TRIF-dependent pathway, activating transcription factors such as NF-κB, AP-1, IRF3 or IRF7. Finally, TLR signaling induces inflammatory cytokines or interferons. (*b*) Schematic representation of TLR8. The ectodomain of TLR8 consists of 26 LRRs and the N-terminal and the C-terminal regions. A characteristic loop, the Z-loop, is inserted between LRR14 and LRR15. A single transmembrane helix and the TIR domain are located on the C-terminus.

Toll-like receptors (TLRs) are a family of single transmembrane receptors that recognize molecular patterns from microbes or viruses and activate the innate immune system (Fig. 1 ▸ *a*). Some receptors recognize pathogen-associated molecular patterns (PAMPs) or damage-associated molecular patterns (DAMPs) to rapidly respond to a wide range of invading agents. These receptors are called pattern-recognition receptors (PRRs) and consist of TLRs, Rig-I-like receptors (RLRs), NOD-like receptors (NLRs) and C-type lectin receptors (CLRs) (Takeuchi & Akira, 2010 ▸). TLRs are type I membrane receptors that are located on cell surfaces or in endosomes. Ten TLRs have been identified in humans, including TLR1, TLR2, TLR4, TLR5 and TLR6, which are located on cell surfaces and mainly recognize the components of bacteria; others, including TLR3, TLR7, TLR8 and TLR9, are located in endosomes and recognize pathogen-derived nucleic acids (Kawai & Akira, 2010 ▸). Currently, no consensus has been reached regarding the function of TLR10, despite several proposed hypotheses.

TLRs typically exist as monomers, and ligand binding causes homodimerization or heterodimerization, transducing extracellular signals to the inside of the cell and finally inducing the production of inflammatory cytokines or interferons (Kawai & Akira, 2010 ▸). TLRs consist of an extracellular (or, to be precise, luminal for the endosomal TLRs) leucine-rich repeat (LRR) domain, a single transmembrane (TM) domain and a cytoplasmic Toll-interleukin-1 receptor homology (TIR) domain (Fig. 1 ▸ *b*; Song & Lee, 2012 ▸). The LRR domains play a role in ligand recognition, and the TIR domains mediate downstream signals, accompanied by adaptor proteins that contain TIR domains, such as MyD88, TIRF, TIRAP/MAL, TRAM or SARM (Kawai & Akira, 2010 ▸). Finally, they activate transcription factors such as NF-κB, AP-1, IRF3 or IRF7, inducing the production of inflammatory cytokines or type I interferons.

Ligand-recognition mechanisms have been revealed by X-ray crystal structural analyses of TLR1/6/triacylated lipopeptide (Jin *et al.*, 2007 ▸), TLR2/6/diacylated lipopeptide (Kang *et al.*, 2009 ▸), TLR3/double-stranded RNA (dsRNA) (Liu *et al.*, 2008 ▸), TLR4/MD-2/LPS (Park *et al.*, 2009 ▸; Ohto *et al.*, 2012 ▸), TLR5/flagellin (Yoon *et al.*, 2012 ▸), TLR7 or TLR8/mononucleosides and single-stranded RNA (ssRNA) (Zhang *et al.*, 2016 ▸; Zhang, Ohto *et al.*, 2018 ▸; Tanji *et al.*, 2013 ▸, 2015 ▸) and TLR9/CpG DNA or single-stranded DNA (ssDNA) (Ohto *et al.*, 2015 ▸, 2018 ▸). TLR10 has been hypothesized to interact with lipopeptides to form dimers with TLR2 in a similar manner to TLR1 or TLR6 and negatively regulate signaling (Hess *et al.*, 2017 ▸); however, its detailed function remains unknown.

From the structural viewpoint, each LRR domain of the TLRs forms a horseshoe-like or ‘c’-shaped structure, and each dimer makes an ‘m’-shaped dimer through the ligand and the C-terminal regions of protomers facing each other. It has been hypothesized that conformational changes in the ectodomains through dimerization cause TIR–TIR interactions (Ve *et al.*, 2017 ▸).

In this review, we focus on TLR8 (Fig. 1 ▸ *b*). Intensive *in vitro* structural and biophysical analyses of TLR8 have been conducted. To date, agonist-induced activated dimer structures, an unliganded dimer structure and antagonist-induced dimer structures have already been reported, and functional mechanisms have been proposed (Tanji *et al.*, 2013 ▸, 2015 ▸; Zhang, Hu *et al.*, 2018 ▸; Hu *et al.*, 2018 ▸). Here, we summarize recent structural studies and discuss the mechanism by which ligand binding influences or regulates the activity of TLR8.

## 2. General features of TLR8   

Among the TLRs, TLR3, TLR7, TLR8 and TLR9 are localized in endosomes and recognize patterns of nucleic acids (Zhang *et al.*, 2017 ▸). It has been reported that a chaperone protein, UNC93B1, regulates the stability and/or transportation of these TLRs. Furthermore, TLR researchers have investigated whether UNC93B1 is related to the regulation of TLR functions (Majer, Liu, Kreuk *et al.*, 2019 ▸; Majer, Liu, Woo *et al.*, 2019 ▸). As TLR7, TLR8 and TLR9 share some common features in sequence homology, function and structure, they constitute the TLR7 subfamily and harbor 26 LRRs, thus being the longest members of the TLR family, sense single-stranded nucleic acids and have a characteristic insertion between LRR14 and LRR15 called the Z-loop (Tanji *et al.*, 2013 ▸; Ohto *et al.*, 2015 ▸; Zhang *et al.*, 2016 ▸; Fig. 1 ▸ *b*). The Z-loop is critically important in their functions as it has been reported that cleavage of the Z-loop and reorganization of the TLR is required for activation, as shown by structural analyses and biochemical experiments (Tanji *et al.*, 2016 ▸). TLR7 and TLR8 resemble each other particularly closely in the TLR family. They have a common function in sensing ssRNA and common ligands, such as the antiviral imidazoquinoline resiquimod R848 (Jurk *et al.*, 2002 ▸). Furthermore, they play a principal role in the response to viral infections mediated by the MyD88-dependent pathway, which activates NF-κB and MAPK and induces the production of inflammatory cytokines such as tumor necrosis factor α (TNF-α), IL-6 and IL-12.

Notably, the differences between TLR7 and TLR8 are their sensing specificity for a sequence of nucleic acids and their expression in cells. While TLR7 preferentially recognizes guanosine and ssRNA containing the UU motif (Zhang *et al.*, 2016 ▸; Zhang, Ohto *et al.*, 2018 ▸), TLR8 recognizes uridine and ssRNA (Tanji *et al.*, 2015 ▸). With regard to expression in cells, TLR7 is expressed at relatively high levels in plasmacytoid dendritic cells, eosinophils, neutrophils and B cells, with TLR8 being expressed in myeloid dendritic cells, neutrophils and monocytes (Marques & Williams, 2005 ▸), suggesting that the function of TLRs is influenced or divided by cell differentiation or localization. Furthermore, recent studies have suggested that TLR7 and TLR8 differentially activate downstream pathways (Marcken *et al.*, 2019 ▸) and that TLR8 is upregulated and works compensatorily in TLR7^−/−^ mice (Awais *et al.*, 2017 ▸).

## 3. Agonist-induced activated forms of TLR8   

**Figure 2:**
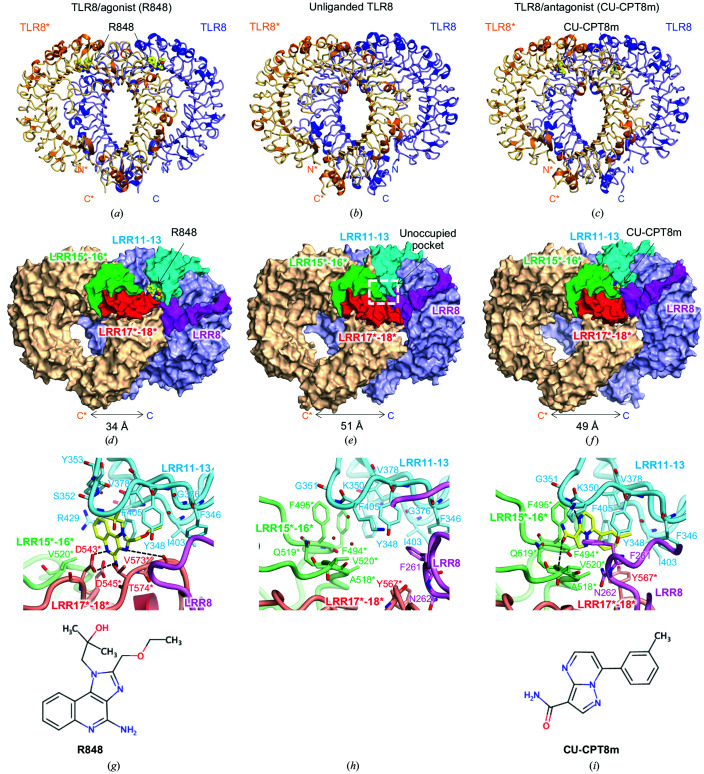
Structures of agonist-bound, unliganded and antagonist-bound forms. (*a*, *b*, *c*) The crystal structures of (*a*) agonist (R848)-bound, (*b*) unliganded and (*c*) antagonist (CU-CPT8m)-bound forms. A representative image of one protomer is colored blue and the other is colored orange and marked with an asterisk (*). The ligands are represented as ball-and-stick models in which C atoms are represented in yellow, O atoms in red and N atoms in blue. (*d*, *e*, *f*) The overall structures of (*d*) agonist-bound, (*e*) unliganded and (*f*) antagonist-bound forms. The leucine-rich repeat (LRRs) around the ligand-recognition sites are highlighted in purple (LRR8), cyan (LRR11–13), green (LRR15*–16*) and red (LRR17*–18*). The unoccupied pocket is shown by a white dashed rectangle. In the agonist-bound structure, the C-terminal regions are closer than those in the inactivated structures. The antagonist-bound structure was similar to the unliganded structure. (*g*, *h*, *i*) Close-up views around (*g*) the agonist-bound site (PDB entry 3w3l), (*h*) the unoccupied pocket (PDB entry 3w3g) and (*i*) the antagonist-bound site (PDB entry 5wyx). The chemical structure of each ligand is shown below the close-up view. Interactions of the agonist involve hydrophobic residues such as Phe346, Tyr348, Gly376, Val378, Ile403–Phe405, Val520*, Asp543*, Gly572*–Thr574* and some hydrogen bonds. Interactions of the antagonist involve hydrophobic residues such as Phe261, Phe346, Val378, Ile403, Phe405, Phe494*, Ala518*, Val520* and Tyr567*, stacking interactions with Tyr348 and Phe495* and some hydrogen bonds. LRR11–13 confront LRR15*–16* in the unliganded structure and the antagonist-bound structure, while LRR11–13 mainly interact with LRR17*–18* in the agonist-bound structure.

In 2013, the crystal structure of the TLR8 ectodomain was reported for the first time in the TLR7 subfamily. Dimeric structures of TLR8 with or without agonistic synthetic imidazoquinoline compounds (R848, CL097 and CL075) were reported (Figs. 2 ▸ *a* and 2 ▸ *b*; Tanji *et al.*, 2013 ▸). These results demonstrated that TLR8 forms an unliganded dimer state, unlike other TLRs localized on cell surfaces, and is converted into an activated dimer state triggered by ligand binding.

The C-terminal regions of the unliganded dimeric structure are separated by ∼50 Å, but the two C-termini are brought into close proximity (∼30 Å) upon ligand binding, which is suitable for association of the intracellular TIR domains (Figs. 2 ▸ *a* and 2 ▸ *b*). This can be explained by rearrangement of the LRRs driven by agonists. The synthetic agonists are bound at symmetric positions between two protomers and interact with the LRRs around LRR11–14 and LRR16*–18* (asterisks are used to distinguish the counterpart protomer) in a different way from the unliganded dimeric structure (Figs. 2 ▸ *d* and 2 ▸ *e*). The ligands create several hydrogen bonds and hydrophobic interactions with the TLR8 residues (Fig. 2 ▸ *g*), and an NF-κB reporter assay with single point mutations of TLR8 demonstrated the importance of Phe405, Asp543, Tyr348, Val520 and Thr574, and the lesser significance of Arg429, Asp545 and Tyr353, in the function and ligand recognition of TLR8.

In addition to synthetic agonist-bound states, the crystal structure of TLR8 complexed with a 20-mer ssRNA has been reported (Tanji *et al.*, 2015 ▸). Unexpectedly, this study revealed that TLR8 was associated with uridine and the RNA fragment derived from an ssRNA at two distinct sites. Uridine is present at the site corresponding to the synthetic agonist-bound site, which is termed the first site. Furthermore, the ssRNA fragment was also found around LRR10–13, which is termed the second site. Although biochemical experiments have indicated that uridine has a lower affinity than that exhibited by the synthesized agonists, the synergetic effect is comparable to that of the synthesized agonists. As similar effects have been observed and verified in TLR7 and TLR9 (Zhang *et al.*, 2016 ▸; Ohto *et al.*, 2018 ▸), the TLR7 subfamily shares the same activation mechanisms.

**Figure 3:**
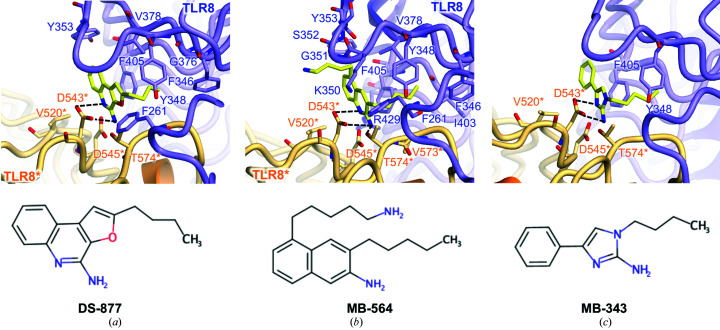
Close-up views of agonist recognition. (*a*, *b*, *c*) Close-up views around the agonist-bound site with (*a*) DS-877 (PDB entry 3wn4), (*b*) MB-564 (PDB entry 5awc) and (*c*) MB-343 (PDB entry 5az5). The agonists are represented as ball-and-stick models in which C atoms are represented in yellow, O atoms in red and N atoms in blue. Hydrogen bonds are indicated using dashed lines. The chemical structure of each ligand is shown below the close-up view.

Based on the accumulated structural information, various agonists have been developed, including TLR7- and TLR8-specific agonists, and TLR7/8 dual ligands (Fig. 3 ▸, Table 1 ▸; Beesu *et al.*, 2015 ▸; Kokatla *et al.*, 2014 ▸; Yoo *et al.*, 2014 ▸; Ganapathi *et al.*, 2015 ▸; Beesu, Caruso *et al.*, 2016 ▸). As the name suggests, the backbone of imidazoaquinoline compounds, which are the most well known TLR8 agonists, consists of tricyclic aromatic rings originating from imidazole and quinoline. Several derivatives have been synthesized to regulate the potency or TLR specificity and have been characterized by structural analyses or biochemical experiments. Similar tricyclic compounds such as DS-877, which includes a furanyl ring, can interact with TLR8 (Fig. 3 ▸ *a*). However, subsequent studies revealed that compounds such as MB-564, with a simpler backbone including an indole ring, were also able to activate TLR8 (Fig. 3 ▸ *b*). Moreover, a subsequent study has demonstrated that characteristic compounds such as MB-343, with an imidazole ring and a phenyl ring, are able to bind to TLR8 as well as other agonists and activate TLR8 (Fig. 3 ▸ *c*).

Structural analyses of TLR8 and various agonists have shown that ligand binding to TLR8 in an agonistic manner does not require interactions with many residues, but critical interactions exist. Tyr348, Phe405, Val520*, Asp543* and Thr574* are always involved in the formation of interaction networks, while cell experiments further indicated the importance of these residues. In particular, Phe405 and Asp543* are located in the proximity of agonists and form stacking interactions or hydrogen bonds, suggesting their critical role in the agonist-recognition mechanism of TLR8.

## 4. Inactivated forms of TLR8 stabilized by antagonists   

In addition to the structures of activated forms of TLR8, TLR8–antagonist complex structures were reported in 2018 (Zhang, Hu *et al.*, 2018 ▸). This study reported CU-CPT compounds as the first human TLR8-specific antagonists, along with structural information in order to understand the TLR8 inhibitory mechanism and accelerate the development of TLR8-targeted medicines with inhibitory effects.

The overall structure of TLR8 with antagonists is essentially the same as that of the unliganded dimeric structure, in which the C-terminal regions are distant from each other (Figs. 2 ▸ *b*, 2 ▸ *c*, 2 ▸ *e* and 2 ▸ *f*). The antagonists were bound at two pockets, created by LRR11–13 and LRR15*–16*, sandwiched at the interface of two protomers. The pocket in the unliganded dimer was partially filled with water molecules (Fig. 2 ▸ *h*). The antagonists formed hydrogen bonds, stacking interactions and hydrophobic interactions with several residues of the LRRs around the pocket, which cause local conformational changes (Figs. 2 ▸ *h* and 2 ▸ *i*). However, this did not induce considerable changes in other regions, and hence it was concluded that the antagonists fix and stabilize the in­activated dimer state almost equivalently to the unliganded dimer state. The antagonists fit into the pocket surrounded by hydrophobic residues such as Phe261, Phe346, Val378, Ile403, Phe405, Phe494*, Ala518*, Gln519*, Val520* and Tyr567*. Stacking interactions with Tyr348 and Phe495* and some hydrogen bonds also contribute to ligand binding.

**Figure 4:**
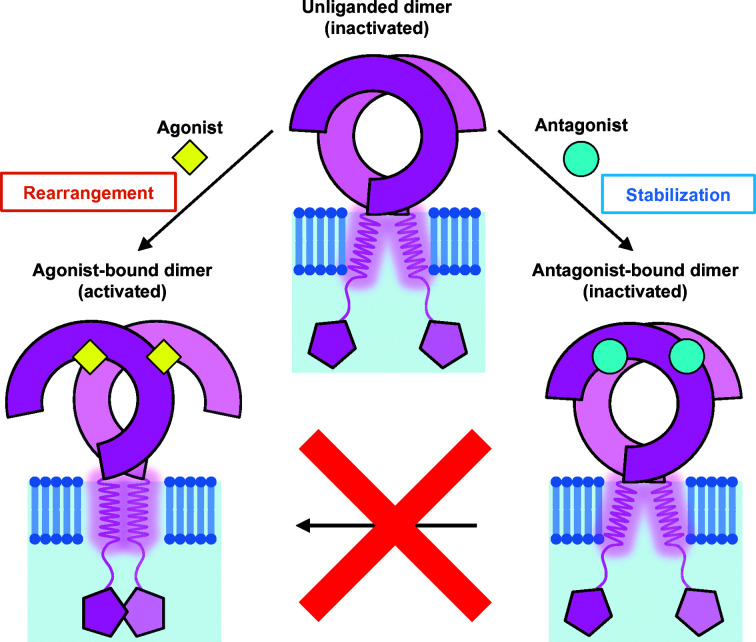
Illustration of the unliganded state and the ligand-induced activated and inactivated states of TLR8. TLR8 forms a dimeric structure without ligands. Agonist binding causes rearrangement of TLR8 into the activated structure. The antagonists fix and stabilize the inactivated structure, which prevents agonists from binding to TLR8. In the illustration the ligands are shown to be present on the TLR8 dimers, but in actuality they are sandwiched between the two protomers.

Biochemical experiments have validated the antagonistic activity of the CU-CPT compounds. Isothermal titration calorimetry experiments showed that agonists such as R848 were unable to bind to TLR8 in the presence of antagonists; in cell experiments, it was confirmed that addition of antagonists significantly reduced the expression of proinflammatory cytokines such as TNF-α or IL-8. The NF-κB reporter assay also indicated that antagonists suppressed R848-induced activation. The occupation of the pockets effectively excludes the intrusion of agonists onto the interface of the TLR8 dimer, switching it to the activated structure (Fig. 4 ▸).

**Figure 5:**
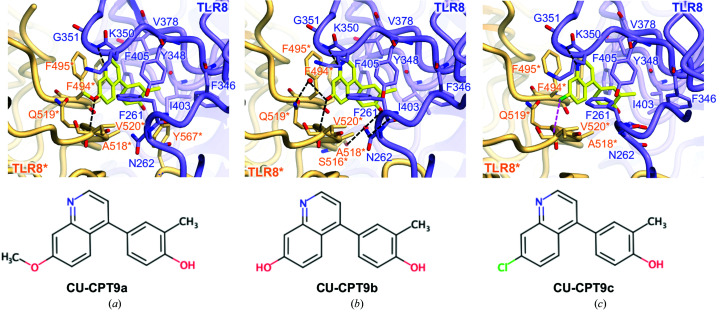
Close-up views of antagonist recognition. (*a*, *b*, *c*) Close-up views around the antagonist-bound site with (*a*) CU-CPT9a (PDB entry 5z14), (*b*) CU-CPT9b (PDB entry 5wyz) and (*c*) CU-CPT9c (PDB entry 5z15). The agonists are represented as ball-and-stick models in which C atoms are represented in yellow, O atoms in red, N atoms in blue and chloride ions in green. Hydrogen bonds are shown as black dashed lines and the halogen bond is shown as a magenta dashed line. The chemical structure of each ligand is shown below the close-up view.

In the original report, a derivative antagonist CU-CPT9b was designed and developed to achieve a greater number of hydrogen bonds (Fig. 5 ▸ *b*; Zhang, Hu *et al.*, 2018 ▸), and other antagonistic compounds were reported and characterized in structural and biochemical experiments (Fig. 5 ▸, Table 1 ▸; Hu *et al.*, 2018 ▸). Furthermore, several compounds consisting of two distinct chemical scaffolds equipped with TLR7/8 dual or TLR8-selective antagonistic activities have recently been discovered (Padilla-Salinas *et al.*, 2019 ▸).

To date, natural antagonists of TLR8 have not been reported. In the case of agonists, the synergetic effect enhances the affinity of uridine, a ligand that widely exists in animals and viruses to build RNA. In the case of antagonists, a similar synergetic recognition mechanism has not been elucidated. Although the inactivated dimer structure with antagonists provided an important clue to understanding the mechanism by which TLR8 can be regulated by antagonists, investigations concerning the inhibitory mechanism of TLR8 have only recently been undertaken.

## 5. Detailed comparison of the activated and the inactivated forms   

The antagonist-bound site is spatially close to the agonist-bound site, but the binding mechanism differs considerably. A comparison of these structures enabled us to understand the regulatory mechanism of TLR8. As shown in Figs. 2 ▸(*d*), 2 ▸(*e*) and 2(*f*), LRR8, LRR11–13 and LRR15*–18* play key roles in ligand recognition. In the unliganded state, TLR8 forms an inactivated dimer structure in which LRR11–13 encounter LRR15*–16* and LRR8 confronts LRR17*–18*, creating an unoccupied pocket at the interface of the two protomers (Fig. 2 ▸ *e*). The antagonists bind to the pocket in this arrangement (Figs. 2 ▸ *e*, 2 ▸ *f*, 2 ▸ *h* and 2 ▸ *i*), mainly interacting with LRR11–13 and LRR15*, even though LRR8 and LRR17*–18* are partially involved in ligand recognition. In contrast, the agonists primarily interact with LRR11–13 and LRR17*–18* (Figs. 2 ▸ *d* and 2 ▸ *g*). One protomer, TLR8*, is pulled up in the direction from the C-terminus to the N-terminus of the counterpart protomer by the agonist, repositioning LRR17*–18* in front of LRR11–13, which allows rotation of the overall structure and makes the C-terminal regions closer.

LRR11–13 is a common platform for both agonist and antagonist recognition, and residues in this region, such as Phe346, Tyr348, Val378 and Ile403–Phe405, interact with both agonists and antagonists. Both agonists and antagonists have aromatic rings that are recognized by TLR8 but are recognized in different modes of action (Figs. 2 ▸ *d*, 2 ▸ *f*, 2 ▸ *g* and 2 ▸ *i*). The aromatic ring in the agonists is stacked with Phe405, enabling the agonists to form hydrogen bonds to Asp543*. In contrast, the aromatic ring in the antagonists stacks with Tyr348 and Phe495*. The differing orientation of the aromatic ring in the bound state is one of the determinants of the activity.

## 6. TLR8 as a therapeutic target   

TLRs play a vital role in the innate immune system, and they have become notable targets for the development of therapies in certain diseases. Currently, many clinical trials investigating TLR ligands are in progress, and a few TLR agonists have been approved (Smith *et al.*, 2018 ▸).

As the innate immune system contains a mechanism to boost the adaptive immune system, TLR ligands are promising candidates for adjuvant therapy. Most adjuvant candidates aim to provide treatments for various tumors (Anwar *et al.*, 2019 ▸). While MPL is one of the approved adjuvants targeting TLR4, another well known and widely used compound is imiquimod, a TLR7 agonist. Imiquimod has been approved by the FDA and is used in various diseases such as external genital and perennial warts, actinic keratosis and non-melanoma skin cancers, and is currently in clinical trials to obtain further indications (Vanpouille-Box *et al.*, 2019 ▸). Resiquimod (R848), a TLR7/8 agonist similar to imiquimod, is a favorable candidate in clinical trials. As a TLR8-selective agonist, VTX-2337, which is proposed to augment antibody-dependent cellular cytotoxicity through activation of NK cells (Lu *et al.*, 2012 ▸), has also been assessed in clinical trials. In addition to these examples, other novel compounds have been successively characterized and reported as candidate adjuvants for targeting TLR8 or TLR7/8 (Yoo *et al.*, 2014 ▸; Beesu, Salyer *et al.*, 2016 ▸; Beesu *et al.*, 2017 ▸).

IMO-8400 is a TLR7/8/9 ligand that is currently being investigated for clinical application in the treatment of immune-mediated inflammatory diseases such as psoriasis. Impressively, IMO-8400 has been reported to be a first-in-class oligonucleotide antagonist that is proposed to suppress aberrant TLR-mediated inflammation (Balak *et al.*, 2017 ▸). This is noteworthy because to date the structural and molecular basis for the antagonistic mechanism of the oligonucleotide for TLR7/8 was unknown, although the inhibition mechanism of TLR9 and the activation mechanism of TLR7/8/9 by nucleoside sensing have been reported (Ohto *et al.*, 2015 ▸; Tanji *et al.*, 2015 ▸; Zhang *et al.*, 2016 ▸). If TLR7/8 directly interacts with oligonucleotides in an antagonistic manner, it will provide a new scheme of TLR regulation at the molecular level.

In terms of pathology, the collapse of TLR8 or other TLRs leads to infection with multiple viruses. Meanwhile, the relationship between TLR8 and autoimmune diseases has received considerable attention (Farrugia & Baron, 2017 ▸), with well known examples including systemic lupus erythematosus (Devarapu & Anders, 2018 ▸) and rheumatoid arthritis (Elshabrawy *et al.*, 2017 ▸). Since TLR8 (and TLR7) senses and responds to various kinds of RNA viruses (Marcken *et al.*, 2019 ▸; Coch *et al.*, 2019 ▸), TLR8 deficiency has been proposed to cause viral infections; however, it has been reported that TLR8 deletion accelerates autoimmunity in mice (Tran *et al.*, 2015 ▸).

Another interesting perspective is the function of TLRs in the nervous system. Notably, roles of TLRs in immunity and neurogenesis in the central nervous system (CNS) have been reported. Recent studies have suggested that TLRs influence neurogenesis, neurodegeneration and neuronal morphogenesis (Barak *et al.*, 2014 ▸; Fiebich *et al.*, 2018 ▸; Chen *et al.*, 2019 ▸), indicating that TLRs are relevant to inflammation and neurodegenerative diseases such as Parkinson’s disease and Alzheimer’s disease. Although TLRs in the CNS may continue to demonstrate unknown functions, elucidating the mechanisms of these functions is an intriguing challenge which will be beneficial for the development of new therapies for CNS diseases.

**Figure 6:**
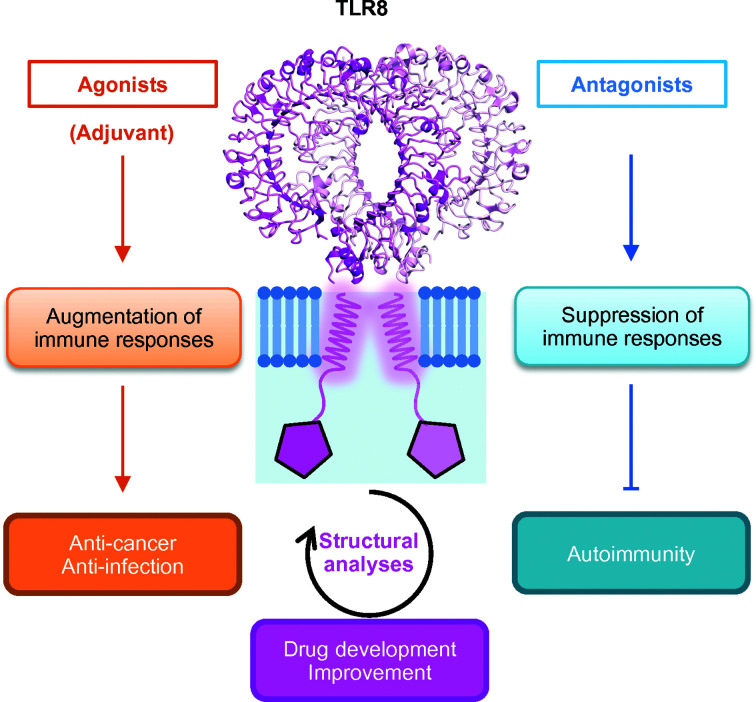
Application for disease treatment. The agonists are mainly used as adjuvants to augment immune responses to cancer or infection. Antagonists are used to suppress aberrant immune responses owing to autoimmunity. Structural characterization helps to develop and to improve the drug candidates.

TLRs are involved in various diseases and physiological phenomena. Some drugs or adjuvants targeting TLRs have already been approved, and other candidates have successively been developed. The development of both agonists and antagonists is currently in progress and has been proposed to establish novel therapies for various cancers or autoimmunity-related diseases (Fig. 6 ▸).

## 7. Concluding remarks   

TLRs are crucial receptors for innate immunity. Previously, structural information on the nucleic acid-sensing TLRs had been limited to TLR3, but the structure of TLR8 was resolved in 2013. Currently, structures are available for all members of the TLR7 subfamily. TLR8 structures showed some characteristic features that are conserved in the TLR7 subfamily, which differs drastically from other subfamilies of TLRs. In addition, ligand-complexed structures of TLRs provide hints to understanding the mechanisms of ligand recognition and signal transduction. Structural information regarding agonist-bound and antagonist-bound TLR8 will accelerate the development of novel therapeutic approaches targeting these TLRs. In particular, the structural information on antagonists may potentially be a paradigm-shifting discovery, even though TLR inhibitor/agonist design has been an active research field, with almost all previous efforts focused on the recognition of activated forms of TLRs.

One remaining concern is whether the dimerization of the ectodomains causes the dimerization of the TIR domains; in this situation, the next concern is how assembly occurs. Structural analyses of full-length TLRs including all domains and complexed with adaptor proteins are required to elucidate the comprehensive mechanism of TLR signaling at the molecular level. This is a challenge for researchers to overcome in structural biology.

## Figures and Tables

**Table 1:** X-ray crystallographic structures of human TLR8

Ligand	Type	Resolution (Å)	PDB code(s)	Reference
(Apo form)	Unliganded	2.3	3w3g	Tanji *et al.* (2013 ▸)
CL097, CL075, R848 (three forms)	Agonist	2.0, 2.3, 2.1–2.7	3w3j, 3w3k, 3w3l, 3w3m, 3w3n	Tanji *et al.* (2013 ▸)
DS-877	Agonist	1.8	3wn4	Kokatla *et al.* (2014 ▸)
DS-802, XG-1-236	Agonist	2.0, 2.1	4qbz, 4qc0	Yoo *et al.* (2014 ▸)
Hybrid-2	Agonist	2.1	4r6a	Ganapathi *et al.* (2015 ▸)
N1-3, N1-4, MB-568, MB-564	Agonist	2.1, 2.1, 2.2, 2.5	5awb, 5awd, 5awa, 5awc	Beesu *et al.* (2015 ▸)
MB-343	Agonist	2.4	5az5	Beesu, Caruso *et al.* (2016 ▸)
CU-CPT8m, CU-CPT9b	Antagonist	2.4, 2.3	5wyx, 5wyz	Zhang, Hu *et al.* (2018 ▸)
CU-CPT9a, CU-CPT9c	Antagonist	2.8, 2.9	5z14, 5z15	Hu *et al.* (2018 ▸)
ssRNA (ORN06, ssRNA40, ORN06S)	Agonist (RNA)	2.0, 2.4, 2.6	4r07, 4r08, 4r09	Tanji *et al.* (2015 ▸)
Uridine	Agonist (RNA)	1.9	4r0a	Tanji *et al.* (2015 ▸)
(Z-loop uncleaved)	Unliganded	2.6	5hdh	Tanji *et al.* (2016 ▸)

## References

[bb1] Anwar, M. A., Shah, M., Kim, J. & Choi, S. (2019). *Med. Res. Rev.* **39**, 1053–1090. 10.1002/med.21553 PMC6587958 30450666

[bb2] Awais, M., Wang, K., Lin, X., Qian, W., Zhang, N., Wang, C., Wang, K., Zhao, L., Fu, Z. F. & Cui, M. (2017). *Front. Immunol.* **8**, 160. 10.3389/fimmu.2017.00160 PMC5316529 28265274

[bb3] Balak, D. M. W., van Doorn, M. B. A., Arbeit, R. D., Rijneveld, R., Klaassen, E., Sullivan, T., Brevard, J., Thio, H. B., Prens, E. P., Burggraaf, J. & Rissmann, R. (2017). *Clin. Immunol.* **174**, 63–72. 10.1016/j.clim.2016.09.015 27876460

[bb4] Barak, B., Feldman, N. & Okun, E. (2014). *Front. Neurosci.* **8**, 272. 10.3389/fnins.2014.00272 PMC4148028 25221470

[bb5] Beesu, M., Caruso, G., Salyer, A. C. D., Khetani, K. K., Sil, D., Weerasinghe, M., Tanji, H., Ohto, U., Shimizu, T. & David, S. A. (2015). *J. Med. Chem.* **58**, 7833–7849. 10.1021/acs.jmedchem.5b01087 PMC4601487 26351878

[bb6] Beesu, M., Caruso, G., Salyer, A. C. D., Shukla, N. M., Khetani, K. K., Smith, L. J., Fox, L. M., Tanji, H., Ohto, U., Shimizu, T. & David, S. A. (2016). *J. Med. Chem.* **59**, 3311–3330. 10.1021/acs.jmedchem.6b00023 26966993

[bb7] Beesu, M., Salyer, A. C. D., Brush, M. J. H., Trautman, K. L., Hill, J. K. & David, S. A. (2017). *J. Med. Chem.* **60**, 2084–2098. 10.1021/acs.jmedchem.6b01860 28146629

[bb8] Beesu, M., Salyer, A. C. D., Trautman, K. L., Hill, J. K. & David, S. A. (2016). *J. Med. Chem.* **59**, 8082–8093. 10.1021/acs.jmedchem.6b00872 27513008

[bb9] Chen, C.-Y., Shih, Y.-C., Hung, Y.-F. & Hsueh, Y.-P. (2019). *J. Biomed. Sci.* **26**, 90. 10.1186/s12929-019-0584-z PMC6827257 31684953

[bb10] Coch, C., Hommertgen, B., Zillinger, T., Dassler-Plenker, J., Putschli, B., Nastaly, M., Kümmerer, B. M., Scheunemann, J. F., Schumak, B., Specht, S., Schlee, M., Barchet, W., Hoerauf, A., Bartok, E. & Hartmann, G. (2019). *Front. Immunol.* **10**, 371. 10.3389/fimmu.2019.00371 PMC6445952 30972055

[bb11] Devarapu, S. K. & Anders, H.-J. (2018). *J. Biomed. Sci.* **25**, 35. 10.1186/s12929-018-0436-2 PMC5898010 29650017

[bb12] Elshabrawy, H. A., Essani, A. E., Szekanecz, Z., Fox, D. A. & Shahrara, S. (2017). *Autoimmun. Rev.* **16**, 103–113. 10.1016/j.autrev.2016.12.003 PMC5290220 27988432

[bb13] Farrugia, M. & Baron, B. (2017). *Int. J. Inflam.* **2017**, 8391230. 10.1155/2017/8391230 PMC5434307 28553556

[bb14] Fiebich, B. L., Batista, C. R. A., Saliba, S. W., Yousif, N. M. & de Oliveira, A. C. P. (2018). *Front. Cell. Neurosci.* **12**, 329. 10.3389/fncel.2018.00329 PMC6176466 30333729

[bb15] Ganapathi, L., Van Haren, S., Dowling, D. J., Bergelson, I., Shukla, N. M., Malladi, S. S., Balakrishna, R., Tanji, H., Ohto, U., Shimizu, T., David, S. A. & Levy, O. (2015). *PLoS One*, **10**, e0134640. 10.1371/journal.pone.0134640 PMC4537157 26274907

[bb16] Hess, N. J., Jiang, S., Li, X., Guan, Y. & Tapping, R. I. (2017). *J. Immunol.* **198**, 699–707. 10.4049/jimmunol.1601335 PMC5225023 27956526

[bb17] Hu, Z., Tanji, H., Jiang, S., Zhang, S., Koo, K., Chan, J., Sakaniwa, K., Ohto, U., Candia, A., Shimizu, T. & Yin, H. (2018). *Cell Chem. Biol.* **25**, 1286–1291. 10.1016/j.chembiol.2018.07.004 PMC6195466 30100350

[bb18] Jin, M. S., Kim, S. E., Heo, J. Y., Lee, M. E., Kim, H. M., Paik, S.-G., Lee, H. & Lee, J.-O. (2007). *Cell*, **130**, 1071–1082. 10.1016/j.cell.2007.09.008 17889651

[bb19] Jurk, M., Heil, F., Vollmer, J., Schetter, C., Krieg, A. M., Wagner, H., Lipford, G. & Bauer, S. (2002). *Nat. Immunol.* **3**, 499. 10.1038/ni0602-499 12032557

[bb20] Kang, J. Y., Nan, X., Jin, M. S., Youn, S.-J., Ryu, Y. H., Mah, S., Han, S. H., Lee, H., Paik, S.-G. & Lee, J.-O. (2009). *Immunity*, **31**, 873–884. 10.1016/j.immuni.2009.09.018 19931471

[bb21] Kawai, T. & Akira, S. (2010). *Nat. Immunol.* **11**, 373–384. 10.1038/ni.1863 20404851

[bb22] Kokatla, H. P., Sil, D., Tanji, H., Ohto, U., Malladi, S. S., Fox, L. M., Shimizu, T. & David, S. A. (2014). *ChemMedChem*, **9**, 719–723. 10.1002/cmdc.201300573 PMC4105021 24474703

[bb33] Liu, L., Botos, I., Wang, Y., Leonard, J. N., Shiloach, J., Segal, D. M. & Davies, D. R. (2008). *Science*, **320**, 379–381. 10.1126/science.1155406 PMC2761030 18420935

[bb23] Lu, H., Dietsch, G. N., Matthews, M. H., Yang, Y., Ghanekar, S., Inokuma, M., Suni, M., Maino, V. C., Henderson, K. E., Howbert, J. J., Disis, M. L. & Hershberg, R. M. (2012). *Clin. Cancer Res.* **18**, 499–509. 10.1158/1078-0432.CCR-11-1625 22128302

[bb24] Majer, O., Liu, B., Kreuk, L. S. M., Krogan, N. & Barton, G. M. (2019). *Nature*, **575**, 366–370. 10.1038/s41586-019-1612-6 PMC6856441 31546246

[bb25] Majer, O., Liu, B., Woo, B. J., Kreuk, L. S. M., Van Dis, E. & Barton, G. M. (2019). *Nature*, **575**, 371–374. 10.1038/s41586-019-1611-7 PMC6856438 31546247

[bb26] Marcken, M. de, Dhaliwal, K., Danielsen, A. C., Gautron, A. S. & Dominguez-Villar, M. (2019). *Sci. Signal.* **12**, eaaw1347. 10.1126/scisignal.aaw1347 31662487

[bb27] Marques, J. T. & Williams, B. R. G. (2005). *Nat. Biotechnol.* **23**, 1399–1405. 10.1038/nbt1161 16273073

[bb28] Ohto, U., Fukase, K., Miyake, K. & Shimizu, T. (2012). *Proc. Natl Acad. Sci. USA*, **109**, 7421–7426. 10.1073/pnas.1201193109 PMC3358893 22532668

[bb29] Ohto, U., Ishida, H., Shibata, T., Sato, R., Miyake, K. & Shimizu, T. (2018). *Immunity*, **48**, 649–658. 10.1016/j.immuni.2018.03.013 29625894

[bb30] Ohto, U., Shibata, T., Tanji, H., Ishida, H., Krayukhina, E., Uchiyama, S., Miyake, K. & Shimizu, T. (2015). *Nature*, **520**, 702–705. 10.1038/nature14138 25686612

[bb31] Padilla-Salinas, R., Anderson, R., Sakaniwa, K., Zhang, S., Nordeen, P., Lu, C., Shimizu, T. & Yin, H. (2019). *J. Med. Chem.* **62**, 10221–10244. 10.1021/acs.jmedchem.9b01201 31687820

[bb32] Park, B. S., Song, D. H., Kim, H. M., Choi, B.-S., Lee, H. & Lee, J.-O. (2009). *Nature*, **458**, 1191–1195. 10.1038/nature07830 19252480

[bb34] Smith, M., García-Martínez, E., Pitter, M. R., Fucikova, J., Spisek, R., Zitvogel, L., Kroemer, G. & Galluzzi, L. (2018). *Oncoimmunology*, **7**, e1526250. 10.1080/2162402X.2018.1526250 PMC6279325 30524908

[bb35] Song, D. H. & Lee, J.-O. (2012). *Immunol. Rev.* **250**, 216–229. 10.1111/j.1600-065X.2012.01167.x 23046132

[bb36] Takeuchi, O. & Akira, S. (2010). *Cell*, **140**, 805–820. 10.1016/j.cell.2010.01.022 20303872

[bb37] Tanji, H., Ohto, U., Motoi, Y., Shibata, T., Miyake, K. & Shimizu, T. (2016). *Proc. Natl Acad. Sci. USA*, **113**, 3012–3017. 10.1073/pnas.1516000113 PMC4801236 26929371

[bb38] Tanji, H., Ohto, U., Shibata, T., Miyake, K. & Shimizu, T. (2013). *Science*, **339**, 1426–1429. 10.1126/science.1229159 23520111

[bb39] Tanji, H., Ohto, U., Shibata, T., Taoka, M., Yamauchi, Y., Isobe, T., Miyake, K. & Shimizu, T. (2015). *Nat. Struct. Mol. Biol.* **22**, 109–115. 10.1038/nsmb.2943 25599397

[bb40] Tran, N. L., Manzin-Lorenzi, C. & Santiago-Raber, M.-L. (2015). *Immunology*, **145**, 60–70. 10.1111/imm.12426 PMC4405324 25424423

[bb41] Vanpouille-Box, C., Hoffmann, J. A. & Galluzzi, L. (2019). *Nat. Rev. Drug Discov.* **18**, 845–867. 10.1038/s41573-019-0043-2 31554927

[bb42] Ve, T., Vajjhala, P. R., Hedger, A., Croll, T., DiMaio, F., Horsefield, S., Yu, X., Lavrencic, P., Hassan, Z., Morgan, G. P., Mansell, A., Mobli, M., O’Carroll, A., Chauvin, B., Gambin, Y., Sierecki, E., Landsberg, M. J., Stacey, K. J., Egelman, E. H. & Kobe, B. (2017). *Nat. Struct. Mol. Biol.* **24**, 743–751. 10.1038/nsmb.3444 PMC8059215 28759049

[bb43] Yoo, E., Salunke, D. B., Sil, D., Guo, X., Salyer, A. C. D., Hermanson, A. R., Kumar, M., Malladi, S. S., Balakrishna, R., Thompson, W. H., Tanji, H., Ohto, U., Shimizu, T. & David, S. A. (2014). *J. Med. Chem.* **57**, 7955–7970. 10.1021/jm500744f PMC4191598 25192394

[bb44] Yoon, S., Kurnasov, O., Natarajan, V., Hong, M., Gudkov, A. V., Osterman, A. L. & Wilson, I. A. (2012). *Science*, **335**, 859–864. 10.1126/science.1215584 PMC3406927 22344444

[bb45] Zhang, S., Hu, Z., Tanji, H., Jiang, S., Das, N., Li, J., Sakaniwa, K., Jin, J., Bian, Y., Ohto, U., Shimizu, T. & Yin, H. (2018). *Nat. Chem. Biol.* **14**, 58–64. 10.1038/nchembio.2518 PMC5726935 29155428

[bb46] Zhang, Z., Ohto, U., Shibata, T., Krayukhina, E., Taoka, M., Yamauchi, Y., Tanji, H., Isobe, T., Uchiyama, S., Miyake, K. & Shimizu, T. (2016). *Immunity*, **45**, 737–748. 10.1016/j.immuni.2016.09.011 27742543

[bb47] Zhang, Z., Ohto, U., Shibata, T., Taoka, M., Yamauchi, Y., Sato, R., Shukla, N. M., David, S. A., Isobe, T., Miyake, K. & Shimizu, T. (2018). *Cell. Rep.* **25**, 3371–3381. 10.1016/j.celrep.2018.11.081 30566863

[bb48] Zhang, Z., Ohto, U. & Shimizu, T. (2017). *FEBS Lett.* **591**, 3167–3181. 10.1002/1873-3468.12749 28686285

