# Targeting the innate immune receptor TLR8 using small-molecule agents. Corrigendum

**DOI:** 10.1107/S2059798320010864

**Published:** 2020-08-25

**Authors:** Kentaro Sakaniwa, Toshiyuki Shimizu

**Affiliations:** a Graduate School of Pharmaceutical Sciences, The University of Tokyo, Hongo, Bunkyo-ku, Tokyo 113-0033, Japan

**Keywords:** Toll-like receptors, innate immunity, structural biology, agonist, antagonist, corrigendum

## Abstract

Three of the figures in the article by Sakaniwa & Shimizu [(2020), *Acta Cryst.* D**76**, 621–629] were incorrectly annotated. Corrected figures are published here.

## Abstract 2

The article by Sakaniwa & Shimizu [(2020), *Acta Cryst.* D**76**, 621–629] is corrected.

In the article by Sakaniwa & Shimizu (2020 ▸) some residue labels were interchanged and hydrogen bonds were wrongly connected in Figs. 2, 3 and 5. In Fig. 2(*g*) the labels for D545* and D543* were interchanged and hydrogen bonds were wrongly connected. In Figs. 3(*a*)–3(*c*) the labels for D545* and D543* were interchanged and hydrogen bonds were wrongly connected. In Fig. 5(*b*) hydrogen bonds were wrongly connected.

Corrected figures are published here, and the main-chain atoms that were not involved in the interaction have been removed for clarity in Figs. 2(*g*)–2(*i*) ▸, Fig. 3 ▸ and Fig. 5 ▸.

## Figures and Tables

**Figure 2:**
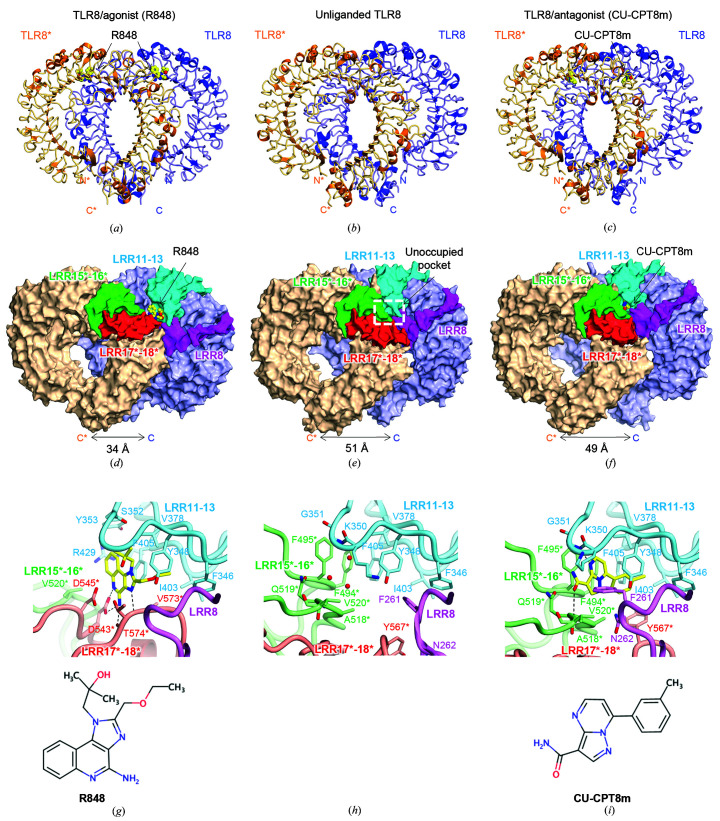
Structures of agonist-bound, unliganded and antagonist-bound forms. (*a*, *b*, *c*) The crystal structures of (*a*) agonist (R848)-bound, (*b*) unliganded and (*c*) antagonist (CU-CPT8m)-bound forms. A representative image of one protomer is colored blue and the other is colored orange and marked with an asterisk (*). The ligands are represented as ball-and-stick models in which C atoms are represented in yellow, O atoms in red and N atoms in blue. (*d*, *e*, *f*) The overall structures of (*d*) agonist-bound, (*e*) unliganded and (*f*) antagonist-bound forms. The leucine-rich repeat (LRRs) around the ligand-recognition sites are highlighted in purple (LRR8), cyan (LRR11–13), green (LRR15*–16*) and red (LRR17*–18*). The unoccupied pocket is shown by a white dashed rectangle. In the agonist-bound structure, the C-terminal regions are closer than those in the inactivated structures. The antagonist-bound structure was similar to the unliganded structure. (*g*, *h*, *i*) Close-up views around (*g*) the agonist-bound site (PDB entry 3w3l), (*h*) the unoccupied pocket (PDB entry 3w3g) and (*i*) the antagonist-bound site (PDB entry 5wyx). The chemical structure of each ligand is shown below the close-up view. Interactions of the agonist involve hydrophobic residues such as Phe346, Tyr348, Gly376, Val378, Ile403–Phe405, Val520*, Asp543*, Gly572*–Thr574* and some hydrogen bonds. Interactions of the antagonist involve hydrophobic residues such as Phe261, Phe346, Val378, Ile403, Phe405, Phe494*, Ala518*, Val520* and Tyr567*, stacking interactions with Tyr348 and Phe495* and some hydrogen bonds. LRR11–13 confront LRR15*–16* in the unliganded structure and the antagonist-bound structure, while LRR11–13 mainly interact with LRR17*–18* in the agonist-bound structure.

**Figure 3:**
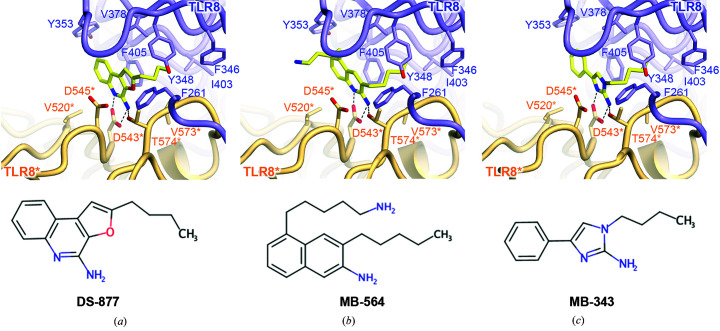
Close-up views of agonist recognition. (*a*, *b*, *c*) Close-up views around the agonist-bound site with (*a*) DS-877 (PDB entry 3wn4), (*b*) MB-564 (PDB entry 5awc) and (*c*) MB-343 (PDB entry 5az5). The agonists are represented as ball-and-stick models in which C atoms are represented in yellow, O atoms in red and N atoms in blue. Hydrogen bonds are indicated using dashed lines. The chemical structure of each ligand is shown below the close-up view.

**Figure 5:**
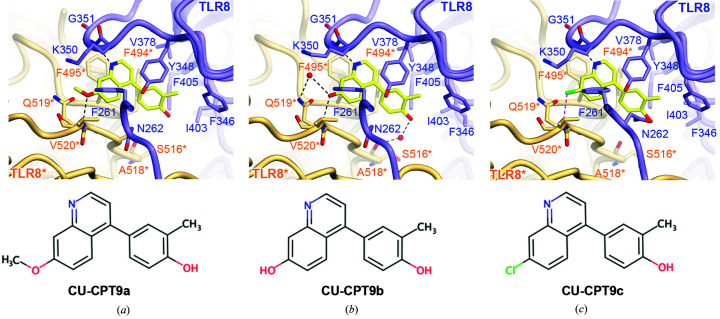
Close-up views of antagonist recognition. (*a*, *b*, *c*) Close-up views around the antagonist-bound site with (*a*) CU-CPT9a (PDB entry 5z14), (*b*) CU-CPT9b (PDB entry 5wyz) and (*c*) CU-CPT9c (PDB entry 5z15). The agonists are represented as ball-and-stick models in which C atoms are represented in yellow, O atoms in red, N atoms in blue and chloride ions in green. Hydrogen bonds are shown as black dashed lines and the halogen bond is shown as a magenta dashed line. The chemical structure of each ligand is shown below the close-up view.
